# Early stress during NICU stay and parent-reported health-related quality of life after extremely preterm birth: an exploratory study with possible targets for early intervention

**DOI:** 10.3389/fped.2024.1381008

**Published:** 2024-04-08

**Authors:** Nienke H. van Dokkum, Arend F. Bos, Karianne E. Kraft, Helene A. Bouma, Sijmen A. Reijneveld, Paul F. M. Krabbe, Marlou L. A. de Kroon

**Affiliations:** ^1^ Divison of Neonatology, Department of Pediatrics, Beatrix Children’s Hospital, University Medical Center Groningen, University of Groningen, Groningen, Netherlands; ^2^ Department of Health Sciences, University Medical Center Groningen, University of Groningen, Groningen, Netherlands; ^3^ Department of Epidemiology, University Medical Center Groningen, University of Groningen, Groningen, Netherlands; ^4^ Environment and Health, Youth Health Care, University of Leuven, KU Leuven, Leuven, Belgium

**Keywords:** health-related quality of life, parental perspectives, extremely preterm infants, neonatal intensive care unit, NICU-related stress

## Abstract

**Introduction:**

The association between neonatal intensive care unit (NICU) related stress in preterm infants and their health-related quality of life (HRQoL) in the first year following preterm birth remains unexplored. Understanding this association is crucial for enhancing preventive and supportive measures for infants and parents within and beyond the NICU.

**Methods:**

From a single center observational cohort study, we included infants with gestational ages below 30 weeks and/or birth weights under 1,000 grams. HRQoL was quantified using the Infant Quality of Life Instrument (IQI) at 3-, 6-, 9- and 12-months corrected age, covering seven domains. NICU stress was quantified using the Neonatal Infant Stressor Scale (NISS) for the first week of life. We performed Spearman's correlation analyses to test this association.

**Results:**

Of the 45 included infants, the IQI was completed for 27 (60%) at 3, 15 (33%) at 6, 14 (31%) at 9 and 15 (33%) at 12 months. The HRQoL sum scores were related to neonatal stress at 9 and 12 months (*ρ* = 0.643 and 0.591, *p* = 0.013 and *p* = 0.019, respectively) but not at 3 and 6 months (*ρ* = −0.001 and −0.077 respectively, *p* > 0.05). Higher NICU stress tended to be associated with more respiratory and mood problems throughout the first year.

**Discussion:**

From a parental perspective on infant HRQoL, extremely preterm infants with higher stress exposure show more problems in the second half-year of life, mainly breathing and possibly mood-related problems. This knowledge may help improve our neonatal care, both during NICU stay and in follow-up clinics, by implementing targeted interventions.

## Introduction

Prematurity is a global health concern that poses a significant challenge. Approximately every one in ten children born worldwide is born preterm, i.e., before 37 weeks of gestation (1, 2). While medical advancements have improved the survival rates of preterm infants, the long-term adverse effects on health and neurodevelopment remain (3–7). For example, these infants are at higher risk of cognitive and motor delays, as well as behavioral and emotional problems. A part of these adverse effects is attributed to exposure to neonatal stress (8), which refers to physiological and psychological stress experiences undergone by preterm neonates during their period in the Neonatal Intensive Care Unit (NICU).

While the developmental consequences of preterm birth have been well established, less is known about health-related quality of life (HRQoL) during the first year of life. Amongst HRQoL domains are regulatory problems, i.e., encompassing feeding, sleeping, and crying. Particularly these three domains have been subject of study and estimates are that approximately one in five preterm children have at least a single regulatory problem (9). The prevalence of combined regulatory problems in crying and sleeping among preterm children is debated, while for feeding a consistently higher prevalence is reported (9). Regulatory problems have consequences in later life, including a higher risk of obesity at pre-school age (10), and childhood attention problems (11). In addition to feeding, sleeping, and crying, parents and experts in the field also consider breathing, stooling, skin, interaction, and mood important domains in the first year after birth (12).

Whether problems in these HRQoL domains are associated with neonatal stress exposure is unknown, but this seems plausible, especially for the regulatory domains (13). Proposed mechanisms include a triggered and exaggerated stress system and a possible impact on parent–child bonding. Understanding the relationship between neonatal stress and HRQoL is crucial for developing more effective preventive and supportive measures for parents and infants both during NICU stay and beyond. Therefore, we aimed to determine the association between early NICU-related stress and outcomes in HRQoL domains during the first year after birth in an exploratory study.

## Methods

### Setting and participants

We included 45 infants as part of the Stress and Outcomes in NICU Graduates (STRONG) study, a single center observational cohort study from the Netherlands. All infants were born between September 2019 and December 2020 and had a gestational age of less than 30 weeks and/or a birth weight below 1,000 grams. All parents of eligible infants were approached shortly after birth and provided written informed consent before inclusion in the study. The study was approved by the Medical Ethical Review Board of the University Medical Center Groningen (METc 2019/128) and this study was also registered online (ISRCTN62164166).

### Quantification of neonatal stress

We prospectively collected all stress-related procedures using the Neonatal Infant Stressor Scale (NISS) from the electronic patient files. This scale is developed by Newham and colleagues to reflect cumulative stress exposure for neonates (14). It includes stressful stimuli in different gradations, i.e., ranging from a little stressful (scored 2 points) to extremely stressful (scored 5 points), and both acute and chronic items are included. Examples of acute stressors include for example suctioning of nose and mouth, lumbar punctions, insertion of intravenous access lines or other skin-breaking procedures. Examples of chronic stressors include for example receiving continuous positive airway pressure (CPAP) or phototherapy or suffering from an infection. All these stressors are summed for a single day to reflect cumulative NICU related stress for that day. We summed each day in the first week of life to form a seven-day cumulative score. A previous study from this cohort showed that most stress occurs in this first week of life (15).

### Quantification of HRQoL

Parents were asked to complete the Infant Quality of Life Instrument (IQI) at 3, 6, 9 and 12 months corrected age. All parents were asked to complete the IQI as part of the cohort study and received e-mail reminders at the indicated time-points. The IQI is a digital application that covers the seven domains of HRQoL deemed essential by parents and experts in the field of pediatrics (12). These domains are feeding, breathing, stooling, sleeping, skin, interaction, and mood. On a scale of one to four, parents were asked to rate whether there are problems on a certain domain and how severe these problems are in their opinion. They did not provide qualitative details, e.g., examples, on the reported problems.

### Statistical analyses

First, we provide descriptive statistics for the cohort of infants. Then, we describe the amount of reported HRQoL problems. As a third step, we summed HRQoL problems and calculated Spearman's correlation coefficients with neonatal stress exposure. Next, we performed more in-depth testing of each domain separately by classifying each domain into 0, not reported as having a problem, and 1, reported to have a problem. In these groups, we compared NISS scores using boxplots and Mann–Whitney *U*-tests.

## Results

In total, 93 infants were eligible to participate in the STRONG study. Of these, 48 did not participate because of missing informed consent due to decease (*n* = 6), language barrier (*n* = 2), or logistical reasons including the COVID-19 research stop (*n* = 25). In total 15 parents declined participation. Of these non-participating infants, some characteristics differed, including being part of a multiple, decease during NICU stay and associated shorter NICU stay and suffering from necrotizing enterocolitis. The final sample consisted of 45 infants, with a median gestational age of 27 weeks (range 26–28 weeks) and a median birth weight of 1,000 grams (range 790–1,248 grams). All participant characteristics are provided in Table 1.

**Table 1:** Participant characteristics for all infants in the “stress and outcomes in NICU graduates study (STRONG)”.

	Descriptive (*N* = 45)
Gestational age (weeks)	27 (26–28)
Birth weight (grams)	1,000 (790–1,248)
Male sex	22 (48.9)
Multiple birth	9 (20.0)
Apgar 1 min	5.5 (3.0–7.0)
Apgar 5 min	7.0 (6.0–8.0)
NICU admission for surviving infants (days)	35 (24–49)
Deceased during NICU admission	2 (0.4)
Delivery via caesarean section	23 (51.1)
Antenatal steroids	38 (84.4)
Complete course	23 (51.1)
IVH grade ≥grade 3	6 (13.3)
Mechanical ventilation	30 (66.7)
Days (*n* = 30)	7.0 (2.8–20.5)
NEC	4 (8.9)
Sepsis	16 (35.6)
Circulatory insufficiency	5 (11.1)
PDA	20 (44.4)

Data are presented as median (25th–75th percentile) or N (%) where appropriate. NICU, neonatal intensive care unit; IVH, intraventricular haemorrhage based on serial cranial ultrasound measurements routinely performed every week; NEC, necrotising enterocolitis; PDA, hemodynamically significant patent ductus arteriosus determined by cardiac ultrasound. Complete course of antenatal steroids was defined as birth >48 h after the first dose. Presence of intestinal pathologies was based on clinical and radiographic examinations. Sepsis was defined as clinical signs of infection, combined with a positive blood culture, and requiring antibiotic treatment. Circulatory insufficiency was defined as requiring fluid therapy and/or treatment with inotropic agent such as dopamine or dobutamine.

### HRQoL domains

**Figure 1:**
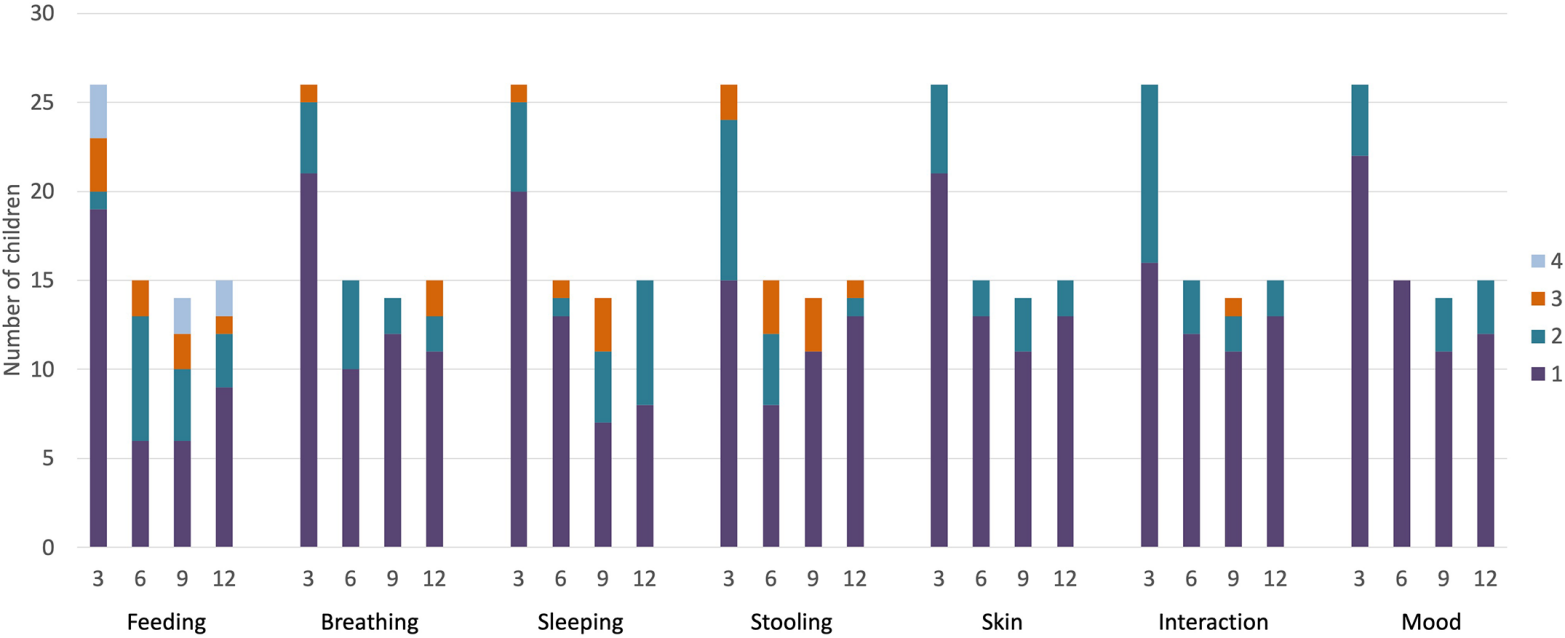
Distribution of parent-reported HRQoL problems for extremely preterm children in the first year after birth. 3 m, 3 months of corrected age; 6 m, 6 months of corrected age; 9 m, 9 months of corrected age; 12 m, 12 months of corrected age. 1, 2, 3 and 4 represent the subsequent levels of problems, ranging from mild to severe.

Of the 45 included infants, the IQI was completed for 27 (60%) at 3 months, 15 (33%) at 6 months, 14 (31%) at 9 months and 15 (33%) at 12 months. Parent-reported problems were most common in the feeding, breathing, sleeping, and stooling domains (Figure 1). In the feeding domain, problems were reported to be most severe.

### Relationship between neonatal stress exposure and HRQoL

**Figure 2:**
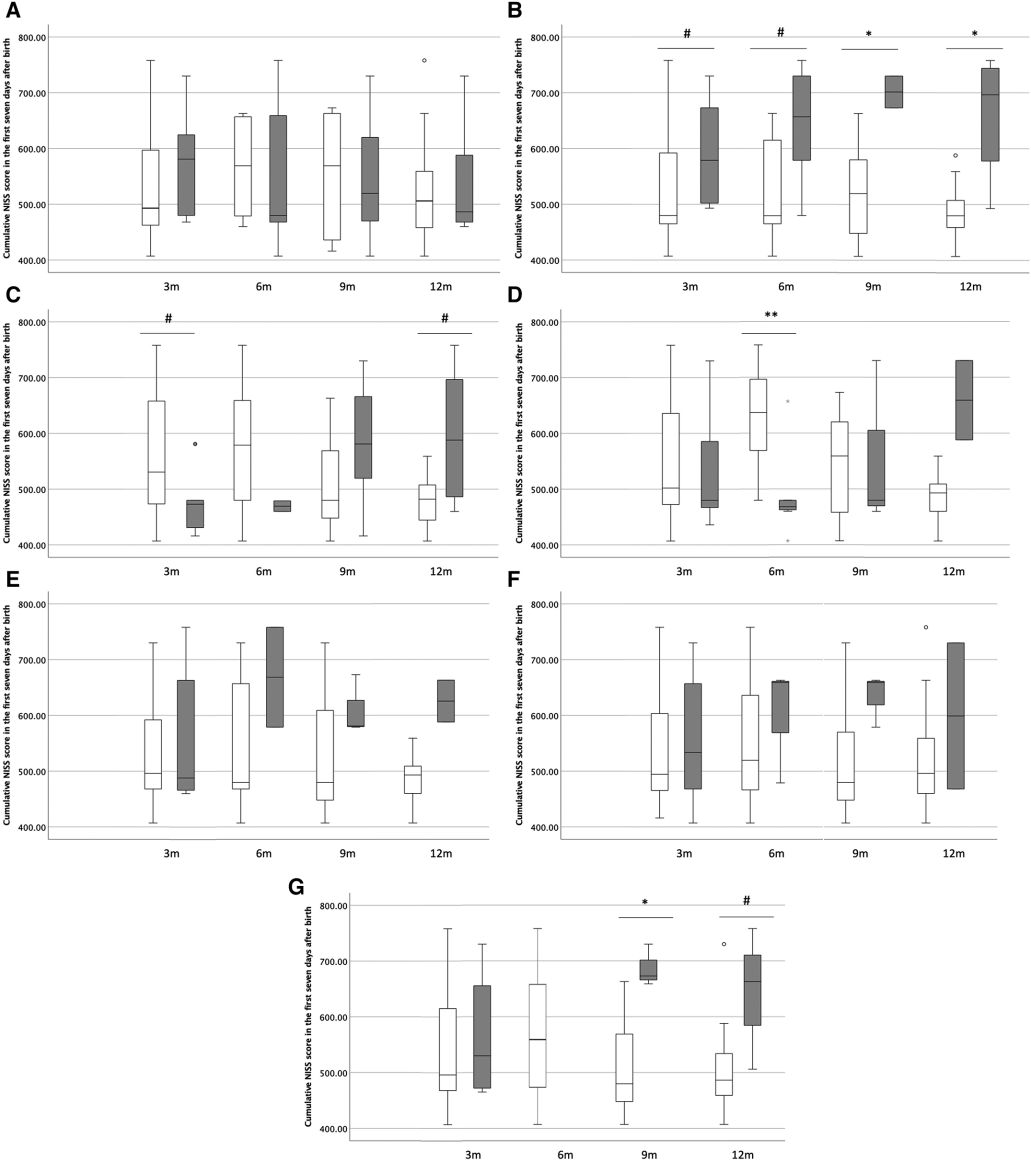
In depth analysis of HRQoL domains in the first year of life. (**A**) Feeding, (**B**) breathing, (**C**) sleeping, (**D**) stooling, (**E**) skin, (**F**) interaction, (**G**) mood. In the box-whisker plots, the boxes represent the distribution of NISS scores of the first seven days after birth (25th to 75th percentile) and the whiskers represent the width of scores. White boxes represent children without parent-reported problems, while grey boxes represent children with parent-reported problems. 3 m, 3 months of corrected age; 6 m, 6 months of corrected age; 9 m, 9 months of corrected age; 12 m, 12 months of corrected age. NISS, Neonatal Infant Stressor Scale. #*p* < 0.10, *p < 0.05, **p < 0.005.

The HRQoL sum scores were not significantly related to neonatal stress exposure at 3 and 6 months of age (*ρ* = −0.001 and −0.077 respectively, *p* > 0.05), whereas at 9 and 12 months of age they were related (*ρ* = 0.643 and 0.591, *p* = 0.013 and *p* = 0.019, respectively). When performing in depth analyses for groups we did not find an association between neonatal stress and feeding problems (Figure 2A). For breathing, we found tendencies towards higher NICU-stress in children with more respiratory problems in the first half year after birth (*p* = 0.085 at 3 months, *p* = 0.057 at 6 months), while we found significant differences in the second half year after birth (*p* = 0.028 at 9 months and *p* = 0.026 at 12 months; Figure 2B). We also found tendencies towards higher NICU-stress in children with more mood problems in the second half year of life (*p* = 0.11 at 9 months and *p* = 0.070 at 12 months; Figure 2G). For the other domains, we did not identify any significant patterns (shown in Figures 2C–F).

## Discussion

With this study, we aimed to determine the association between early NICU-related stress and outcomes in HRQoL domains during the first year after birth. We found that overall neonatal stress exposure during the first seven days after birth are related to cumulative HRQoL problems in the second half-year, but not the first half-year after birth. Additionally, with this exploratory study, we suggest that early neonatal stress is associated with breathing problems, and possibly with more mood problems as well, but not with feeding or sleeping problems, nor with the other HRQoL domains.

We were surprised by our finding of an association between cumulative HRQoL problems and neonatal stress in the second half-year of life, but not in the first half-year of life. To the best of our knowledge, ours is the first study to explore the relationship between NICU-related stress exposure and HRQoL in the first year after birth. We therefore are unable to compare our results to others. We speculate that the second half-year of life includes a transformation from tiny baby to infant, including transitions in several of these HRQoL domains. This may lead to increased parent-reported problems in children exposed to more NICU-related stress, who may handle such transitions differently than children exposed to less NICU-related stress. A mechanism may be altered brain development, or altered stress system development, which we know to occur after higher NICU-related stress exposure (8).

For the individual domains, we found the most consistent differences in stress exposure for children with and without breathing problems throughout the first year after birth. We propose that this is partly because the NISS score includes respiratory support, including conventional ventilation, but also CPAP or high flow nasal cannula (HFNC) support (14). Children with higher stress exposure may therefore have been exposed to more respiratory support. However, this study only included the first seven days after birth as a source of stress exposure and did not look beyond. An additional explanation may regard the existence of bronchopulmonary dysplasia (BPD) in extremely preterm children, who may indeed experience more respiratory problems later in life (16). A third explanation derived from clinical practice may be that children are attending daycare in the second half-year, even though it is advised to parents of preterm children not to in the first year of life. We propose a combination of these explanations underlying our findings.

Importantly, respiratory support and respiratory problems in the first year of life may be improved by early interventions. While medical advancements have already employed many of these, including for example early caffeine treatment and altered ventilation practices, non-pharmacological interventions that can be continued beyond the NICU should not be overlooked. Music therapy may be a promising non-pharmacological intervention in this respect. While studies on music therapy in the first year of life are scarce, we do know that breathing exercises, singing, and playing wind instruments at school age are associated with improvements in asthma symptoms (17). The Rhythm Breath and Lullaby method that is adopted more and more in NICUs worldwide also includes interventions focused on infant breathing. As we also observed more reported mood problems in the second half-year of life in infants with higher NICU-related stress exposure, music therapy may also have implications for that HRQoL domain.

This study included HRQoL from a parental perspective. We strongly believe that the parental perspective is invaluable for follow-up care of our most vulnerable patient population. As we know that parents of extremely preterm infants have altered attachment and bonding (18), and may also experience post-traumatic stress during NICU stay and beyond (19), which may affect their perspectives on health problems in their child, integrating parental perspectives of HRQoL is crucial. Besides including the parental perspective, post-traumatic stress during NICU stay and beyond may also affect physiological development of preterm infants and thereby explain our results. Parental resilience and support by other health-care professionals, even in the home setting, may be part of the explanation of our results too. Perhaps interventions are provided in the first months after discharge that are gradually are phased out in the second half-year of life, reflecting the associations found in our study. While these explanations are only speculative, they should be subject of future studies, just as parenting styles, that may impact regulatory problems and thus HRQoL as well (13). By including the parental perspective, follow-up clinics and professionals working in neonatal follow-up are given a tool to start the conversation directed towards individual cases.

### Strengths and limitations

To our knowledge, this is one of the first studies to take a close look at HRQoL in the first year of life, including neonatal stress as a specific risk factor. As stated before, we consider the parental perspective that is provided in this study as a strength as well. We acknowledge that our study includes a limited sample of extremely preterm infants and that response rates at later timepoints even make the sample smaller. Findings may either be overestimated in the case that parents will respond more promptly if they experience problems, or underestimated in the case that parents experience more space for study participation when they experience little problems. Therefore, results should be interpreted with caution and should be confirmed in larger prospective studies.

## Conclusion

From a parental perspective on HRQoL in seven domains during the first year after birth, extremely preterm infants with higher stress exposure show more problems in the second half-year of life, in particular breathing problems and to a lesser degree mood-related problems. This knowledge may help to improve our neonatal care, both during NICU stay and in follow-up clinics, using tailored interventions.

## Data Availability

The raw data supporting the conclusions of this article will be made available by the authors, without undue reservation.
